# Effect of cognitive and executive functions on perception of quality of life of cognitively normal elderly people dwelling in residential aged care facilities in Sri Lanka

**DOI:** 10.1186/s12877-018-0937-6

**Published:** 2018-10-24

**Authors:** Madushika Wishvanie Kodagoda Gamage, Chandana Hewage, Kithsiri Dedduwa Pathirana

**Affiliations:** 1 Department of Nursing, Faculty of Allied Health Sciences, University of Ruhuna, Galle, Sri Lanka; 2 Department of Physiology, Faculty of Medical Sciences, University of Sri Jayewardenepura, Gangodawila, Nugegoda, Sri Lanka; 3 Department of Medicine, Faculty of Medicine, University of Ruhuna, Galle, Sri Lanka

**Keywords:** Cognition, Working memory, Inhibitory control, Quality of life, Elderly

## Abstract

**Background:**

Although cognitive functions affect the health related quality of life (QoL), the relationship between perceived QoL and cognition including executive functions has not been studied adequately. Available studies show moderate to weak correlations. We evaluated the association of cognition and executive functions, namely working memory (WM) and inhibitory control (IC) with the perceived QoL of a sample of elderly people dwelling in residential aged care facilities (RACFs) in Southern Province of Sri Lanka.

**Methods:**

Cognition was assessed using Mini-Mental State Examination (MMSE), while verbal WM (VWM), visuo-spatial WM (VSWM) and IC (interference control, inhibition of pre potent and ongoing responses) were assessed using VWM, VSWM tasks, colour word Stroop (CWS), go/no-go (GNG) and stop signal (SS) tasks respectively. WHOQoL-Bref (Total score and domain scores) were used to assess QoL. The relationship was analysed using Pearson correlation and hierarchical multiple regression analysis.

**Results:**

Study included 237 elderly people with a mean age of 71.11 ± 6.44 years. Participants scored the highest in the domain of environment (63.48 ± 10.63) and lowest in the domain of social relationships (55.43 ± 21.84) of QoL. Psychological health domain positively correlated with MMSE, VSWM and VWM scores and negatively correlated with CWS, SS and GNG task errors. Both physical health domain and total QoL demonstrated positive correlations with MMSE, VSWM and VWM scores, while negative correlations were observed with CWS task errors. Social relationships domain demonstrated a significant positive correlation with VSWM score. Environment domain positively correlated with MMSE, VSWM and VWM scores and negatively correlated with CWS and SS task errors. All were significant but weak correlations. When controlled for covariates, such as educational status, physical activity and marital status, cognition was a predictor of the domain of environment of QoL, while executive functions were not predictors of total QoL and domains of QoL.

**Conclusion:**

Cognition and executive functions weakly but significantly correlated with different domains of QoL. Only the level of cognition measured by MMSE was a predictor of the domain of environment of QoL and executive functions were not predictors of total QoL and domains of QoL in elderly people with normal cognitive functions dwelling in RACFs.

## Background

Population ageing is a characteristic of the twenty-first century. It is estimated that the proportion of elderly in the population will reach 16.5% and 7.5% in developed and developing countries respectively by 2025 [1]. Sri Lanka is regarded as one of the fastest ageing countries in the world. Although caring for older people is regarded as a moral obligation of children, socio-demographic changes such as increase in proportion of women who engage in employment, decline in number of offspring due to decline in fertility rate, migration of youth and conversion of extended families into nuclear families have resulted in reduction of elderly care [2]. With this, the number of elderly people moving to residential aged care facilities (RACFs) is increasing [2]. These facilities are available in the country for several decades [2]. Meals, accommodation, recreation, protection and other facilities for the residents are provided free of charge and are sponsored by the government throughout the country. Out of 447 elderly care facilities available in Sri Lanka, only 300 (67.1%) are RACFs, while others provide day care [3]. In Sri Lanka, the reason for the provision of residential care is not due to health problems, such as dementia or disability, but due to the lack of infrastructure and availability of personnel to provide care in the community. Having fewer children, the demands of formal sector employment of their children and changing values are the main reasons for their admissions to care facilities [4].

World Health Organization QoL (WHOQoL) Group [5] defined QoL as “individuals’ perceptions of their position in life in the context of the culture and value systems in which they live and in relation to their goals, expectations, standards and concerns”. The individuals’ perception will be affected via their physical health status, personal beliefs, psychological status, social relationships and interaction with the environment. Perception of their position in life is an important aspect for the well-being of the elderly.

Cognition is a process by which “sensory inputs are transformed, reduced, elaborated, stored, recovered and used” [6]. Cognitive health promotion, that is, maintaining “brain health” with ageing has become increasingly important for the elderly [7]. Identification of specific cognitive processes that may underlie cognitive decline is essential for planning preventive measures. Studies are still inconclusive as to whether all components of the nervous system demonstrate a similar degree of age-related changes or whether the effect selectively affects specific brain regions/systems. One such system that has attracted research is prefrontal cortex area-mediated executive functions [8].

Executive functions (EFs) consist of higher order cognitive processes important for goal directed behavior [9]. Working memory and inhibition are regarded as two core processes in EFs [10]. WM is a cognitive system which allows temporarily maintenance of information and manipulation for generating and executing complex activities [11]. It includes a visuo-spatial sketchpad and an articulatory loop, which holds and manipulates visual images including spatial relationship (visuospatial WM) and speech-based information (verbal WM), respectively [12]. Inhibition is the process which regulates information that enters and leaves the WM [13]. Inhibition consists of the ability to overcome interference (protecting a response from disruption by competing responses or events), suppression of pre-potent responses (a response that is or has been previously associated with reinforcement) or stopping of ongoing responses which allows for a delay in the decision to continue responding [14]. Decline in EFs observed with ageing has been associated with significant limitations of functionality, independent living [15] and impaired health enhancing behaviour [16] leading to reduction in QoL of elderly [17]. Therefore “promotion of successful cognitive and emotional ageing” that minimizes loss of information processing capacity and maintains cognitive reserve for elderly, is an important aspect that has to be addressed with population ageing [18].

Although there is a growing interest on assessing effect of cognition on QoL among elderly, currently only a few studies have focused their attention on the relationship between cognition and QoL [19]. Most of the previous studies have focused their attention on association between health related QoL and cognition [20, 21] but not with perception of QoL. Furthermore, the effect of cognition, including EFs on the QoL dimensions, had been inconsistent among different studies [17, 19]. One study revealed significant correlations between MMSE score and QoL domains as physical, environment and overall QoL and not with psychological and social relationships [19], while another revealed significant correlation only between environment domain of QoL and MMSE score [17]. Although they were significant, they demonstrated weak correlations [17, 19]. This reflects that there would be other strong factors that influence QoL. Previous studies have demonstrated the influence of physical activity [22], educational status [23] and marital status [24] on QoL of elderly people. Hence, we thought to identify the association between cognitive and executive functions and QoL when controlled for the factors as physical activity, educational status and marital status. As elderly population is increasing, it is necessary to have a better understanding of the influence of specific neural sub-systems, like EFs, on cognitive decline and its effect on the QoL among elderly. In Sri Lankan culture elderly care is unique and cannot be compared to similar studies. This will enable planning therapeutic interventions in the future.

## Methods

### Study design and participants

**Fig. 1:**
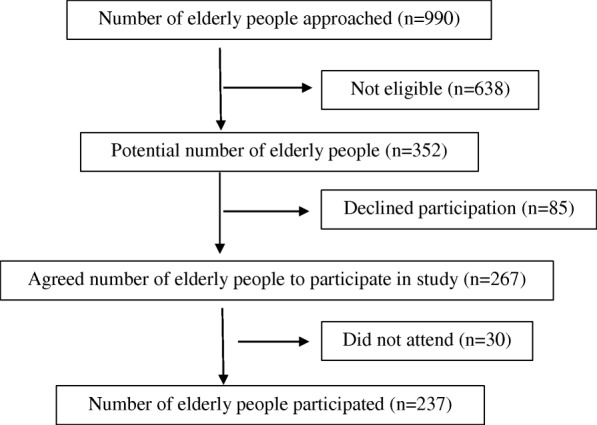
flow chart 1- Recruitment of subjects

The study sample consists of 237 elderly people who are 60 years and older, dwelling in residential aged care facilities in Galle and Matara Districts in Sri Lanka, recruited using probability proportional to size sampling method. During the process of recruitment, an aged care facility was selected randomly. All the elderly people were screened and those who fulfilled the selection criteria and who volunteered to participate were recruited as the study sample in the selected institution. Recruitment of subjects was performed as shown in Fig 1.

Previous literature has shown significant declines in QoL in people with mild cognitive impairment [25]. Hence, we investigated the effect of cognitive and executive functions on QoL of those who have apparently normal cognition. For this purpose, subjects with conditions that affect communication ability, physical activity and cognition were excluded as they affect test performance and they themselves will be confounding factors.

We excluded the subjects with severe loss of vision (corrected vision worse than 6/60), loss of hearing (interviewer-rated), loss of communication ability (interviewer-rated), impaired colour vision, impaired ability to read, write and to follow verbal instructions, subjects with major physical disabilities, who scored less than 100 in Barthel’s index and subjects with conditions that affect performance of tasks, such as stroke, osteoarthritis, amputation, fractures, neurological disorders, subjects with psychiatric illnesses, developmental disabilities and cognitive impairment (MMSE score less than 24).

Demographic characteristics of the participants were obtained using a questionnaire. Physical activity level was assessed using International Physical Activity Questionnaire (IPAQ) modified for elderly version. It provides continuous scores as well as categorical values. Based on their physical activity score, they were categorized as inactive, minimally active and as having health enhancing physical activity level [26].

### Global cognitive measures- mini mental state examination (MMSE)

It is a brief 30-point scale mental health examination which assessed five areas: orientation, registration, attention and calculation and recall and language.

### Core components of EFs- WM and response inhibition

The two core EFs, WM and inhibition were assessed using computerized tasks. Working memory was assessed using verbal WM (VWM) and visuo-spatial WM (VSWM) tasks. Response inhibition was assessed using colour word stroop (CWS), stop signal (SS) and go/no-go (GNG) tasks. All participants were individually tested in a quiet room. The order of task administration was the same for all participants and they received a practice session prior to all the tasks. A period of rest was given between two tasks.

Colour word Stroop task [27, 28]: In this task different colour words appeared on the computer screen one at a time. The task was to name the colour the word was printed, disregarding what the colour word reads. The colour of the word printed was in the same colour as the meaning of the word (congruent trials, eg; “red” is printed in red colour), or it was different from the meaning (incongruent trials, eg; word “green” is printed in blue colour). There were 75 congruent trials and 25 incongruent trials for one test session. Incorrect responses on incongruent trials were taken to assess the level of inhibitory control. The higher the errors the lower the interference control is.

Visuo-spatial WM task: A 4 × 4 matrix with 16 squares was displayed on the computer screen as a pig house with a pig appearing in each window one at a time. The task was to recall in reverse order the locations where each target (pig) had appeared. The test started with a span length of two, that is, two pigs appeared one after another. Each span consisted of two trials and the test was concluded when the participant failed both trials at that same span length. Each correct location was given one point with a maximum score of 88. The score was taken as the measure of VSWM. At the end of the test, obtained score was automatically displayed on the computer screen.

Stop signal task: It assessed the ability to inhibit ongoing responses. This was like a car game [29] where a car appeared on the computer screen. Every time the car appeared, the participant was supposed to press a designated key as fast as possible to drive the car away. But when a stop-sign board appeared next to the car, participants had to refrain from pressing for the car to stand still. Each session in this task consisted of 24 trials with six stop-signs-trials. Number of incorrect presses in stop sign (commission errors) was considered as the measure of inhibition and it was automatically displayed on the screen at the end of the task.

Verbal WM task (adapted from [30]): These were power point slides. Each slide had different numbers of red circles with squares as distracters. The task was to count the total number of red circles in each slide, keep total in memory and recall the numbers in the correct order. The test started with a length of memory recall (span) of two, that is, the participant had to recall two slides first. Each level of memory recall consisted of three trials and the test was concluded when the participant failed two trials out of three at that same length of recall. If the participant was successful in 2 out of three trials, he/she was allowed to go to the next span. A total score was calculated after adding a mark for each correct recall [30].

Go/no-go task (two versions: colour and shape): It assessed ability to inhibit pre potent responses. The subject was presented with four different stimuli on the screen, one at a time in random order. There were two squares and two circles in blue and red. In the first session, the subject was instructed to respond by pressing a key each time when a blue figure appeared (go-trials) regardless of the shape, and not to respond when a red figure appeared. In the second session, the subject was instructed to respond each time when a square appeared, regardless of the color, and not to respond when a circle appeared [31]. Together the two consecutive sessions included 60 stimuli with 77% go-trials. The number of incorrect responses (commission errors) was used as a measure of inhibition and it was automatically displayed on the screen at the end of the task.

QoL was assessed using WHOQoL-Bref short version questionnaire. It measured the perception of an individual about his/her QoL. It contained a subset of 26 items taken from the 100 item questionnaire. It produced a profile with four domain scores which were physical, psychological, environment and social relationships and two individually scored items about an individual’s overall perception of QoL and health. In the questionnaire, the question “How satisfied are you with your sex life?” was omitted from the analysis as all the participants responded either as no or were reluctant to respond.

Domain score, which is a collection of obtained scores for the questions relevant to one domain, was obtained, and it was transformed to a percentage score using the formula shown below. Additionally, total score was calculated reflecting the total QoL.

$$ \mathrm{Transformed}\ \mathrm{scale}=\frac{\left(\mathrm{Actual}\ \mathrm{raw}\ \mathrm{score}\hbox{-} \mathrm{lowest}\ \mathrm{possible}\ \mathrm{raw}\ \mathrm{score}\right)\ast 100}{\mathrm{Possible}\ \mathrm{raw}\ \mathrm{score}\ \mathrm{range}} $$

### Statistical analysis

Statistical analyses were performed using SPSS 20.0 version. The statistical significance was kept at *p* < 0.05. Descriptive analysis was performed to calculate distribution measures. To assess the correlation among QoL and cognitive variables, Pearson correlation test was used, following the classification of Cohen [32], which considered a correlation as weak if *r* < 0.3; moderate if 0.3 ≤ *r* < 0.5 and strong if 0.5 ≤ *r* ≤ 1.0. The variables, such as, educational status, physical activity level and marital status were included as covariates in evaluation of the effect of cognitive and executive functions on QoL. For each of the domains, only the significantly correlated variables were considered as covariates. For physical health domain and total QoL, educational status, physical activity level and marital status were considered as covariates. For psychological health domain, educational status and physical activity were considered as covariates. For environment domain, marital status and physical activity were the covariates while for social relationships, only the educational status was considered as a covariate. Hierarchical multiple linear regression analysis was conducted including covariates into Block 1 and cognitive and executive function scores into Block 2. For block 2, only the cognitive and executive function scores that significantly correlated with QoL domain scores were included.

## Results

The mean age of the participants was 71.11 ± 6.44 of which 63.7% were females. The socio-demographic characteristics are tabulated in Table 1. Most of them were in the age category of more than 70 years, had obtained upper secondary, advanced level and higher education and were married. The participants had the highest score in the domain of environment of QoL and the least score in the domain of social relationships of QoL. Female participants scored higher than male participants in all the domains of QoL except psychological health.

**Table 1:** Socio-demographic characteristics and clinical characteristics of the participants

Characteristic	All elderly (*n* = 237)		Female (*n* = 151)		Male (*n* = 86)	
	N	(%)	N	(%)	N	(%)
Age						
≤ 70 years	108	45.6	79	52.3	29	33.7
> 70 years	129	54.4	72	47.7	57	66.3
Education						
Primary and lower secondary education	101	42.6	67	44.4	34	39.5
Upper secondary, advanced level and higher education	136	57.4	84	55.6	52	60.5
Marital status						
Married	96	40.5	57	37.7	39	45.3
Unmarried	88	37.1	56	37.1	32	37.2
Widowed/ Divorced/Separated	53	22.4	38	25.2	15	17.4
Chronic diseases						
No diseases	87	36.7	53	35.1	34	39.5
1	86	36.3	59	39.1	27	31.4
≥ 2	64	27	39	25.8	25	29.1
Physical activity						
Inactive	3	1.3	2	1.3	1	1.2
Minimally active	169	71.3	101	66.9	68	79.1
Health Enhancing physical activity	65	27.4	48	31.8	17	19.8

Male participants performed a higher number of errors in response inhibition tasks and obtained lower scores in VWM and VSWM tasks than female participants. The mean scores of the MMSE, EF tasks and QoL domains in WHOQoL Bref are presented in Table 2. Table 3 indicates the correlation between QoL domains with MMSE score, WM tasks scores and inhibitory tasks errors. Psychological health domain positively correlated with MMSE (*r* = 0.18,*p* = 0.006), VSWM (*r* = 0.17, *p* = 0.007) and VWM (*r* = 0.15, *p* = 0.021) scores and negatively correlated with CWS (*r* = − 0.14, *p* = 0.03), SS (*r* = − 0.13, *p* = 0.037) and GNG (*r* = − 0.13, *p* = 0.048) task errors.

**Table 2:** Mean score of the MMSE, EF tasks and QoL domains

Task	All elderly	Female	Male	*p*
	Mean (SD)	Mean (SD)	Mean (SD)	
MMSE score	26.81 (± 1.88)	26.95 (± 1.88)	26.57 (± 1.87)	0.13
CWS task incorrect readings	8.62 (± 3.83)	8.13 (± 3.68)	9.49 (± 3.96)	0.008**
SS task incorrect presses	1.83 (± 1.24)	1.76 (± 1.27)	1.95 (± 1.19)	0.24
GNG task incorrect presses	1.11 (± 1.26)	0.98 (± 1.18)	1.32 (± 1.36)	0.053
VSWM task score	12.67 (± 5.44)	12.86 (± 5.52)	12.33 (± 5.33)	0.47
VWM task score	4.29 (± 1.77)	4.39 (± 1.79)	4.13 (± 1.73)	0.27
Physical health domain	62.82 (± 13.94)	63.34 (± 13.21)	61.92 (± 15.16)	0.45
Psychological health domain	59.60 (± 14.08)	59.52 (± 13.86)	59.74 (± 14.54)	0.90
Environment domain	63.48 (± 10.63)	63.80 (± 10.03)	62.90 (±11.65)	0.53
Social relationships domain	55.43 (± 21.84)	55.96 (± 20.51)	54.51 (±22.68)	0.61
Total QoL	59.46 (± 10.54)	59.82 (± 10.56)	58.83 (±10.53)	0.49

*SD* Standard deviation, Significance value *p* < 0.01; **

**Table 3:** Correlation between cognitive variables and domain scores of QoL

Task	Domain	Physical health	Psychological health	Environment	Social relationships	Total QoL
		*R*	*R*	*R*	*R*	*R*
MMSE		0.27**	0.18**	0.29**	0.09	0.25**
VSWM task score		0.27**	0.17**	0.18**	0.15*	0.25**
VWM task score		0.21**	0.15*	0.22**	0.08	0.19**
CWS task errors		−0.26**	−0.14*	−0.18**	−0.02	−0.21**
SS task errors		−0.12	−0.13*	−0.19**	−0.04	−0.11
GNG task errors		−0.01	−0.13*	−0.12	−0.02	−0.07

Significance value *p* < 0.05; *, *p* < 0.01; **

Both physical health domain and total QoL demonstrated positive correlations with MMSE (*r* = 0.27, *p* < 0.001; *r* = 0.25, *p* < 0.001 respectively), VSWM (*r* = 0.27, *p* < 0.001; *r* = 0.25, *p* < 0.001 respectively) and VWM (*r* = 0.21,*p* = 0.001; *r* = 0.19,*p* = 0.004 respectively) scores while negative correlations were observed with CWS (*r* = − 0.26, *p* < 0.001; *r* = − 0.21, *p* = 0.001 respectively) task errors. Social relationships domain demonstrated a significant correlation only with VSWM score (*r* = 0.15, *p* = 0.023) and it was a positive correlation. Environment domain positively correlated with MMSE (*r* = 0.29, *p* < 0.001), VSWM (*r* = 0.18, *p* = 0.006) and VWM (*r* = 0.22, *p* < 0.001) scores and negatively correlated with CWS (*r* = − 0.18, *p* = 0.006) and SS (*r* = − 0.19, *p* = 0.003) task errors. All were weak significant correlations.

Hierarchical multiple linear regression analysis predicting total QoL and QoL domain scores are shown in Table 4. In the domain of physical health, the introduction of cognitive and executive functions explained an additional 5.7% of variance after controlling for covariates (F (7, 229) = 6.97; *p* < 0.001). Physical activity level was the statistically significant variable (*p* < 0.05). Introduction of cognitive and executive functions explained only an additional 7.8% of variance after controlling covariates (F (7, 229) = 5.26; *p* < 0.001) in the domain of environment of QoL. MMSE score was the statistically significant variable (*p* = 0.005). Introduction of cognitive and executive functions did not make a significant difference in variance in total QoL and in the domains of psychological health and social relationships of QoL.

**Table 4:** Hierarchical multiple linear regression predicting total QoL and QoL domain scores

Variable	R	Adjusted R^2^	R^2^ change	Beta	t	*p*
Physical Health domain						
Block 1	0.345	0.108				
Physical activity				.263	3.909	< 0.0001***
Marital status				−.035	−.544	0.59
Educational status				.141	2.187	0.03*
Block 2	0.419	0.150	0.057**			
Physical activity				.174	2.503	0.01**
Marital status				.012	0.186	0.85
Educational status				.091	1.420	0.16
MMSE score				.121	1.781	0.08
VSWM task score				.104	1.453	0.15
VWM task score				.042	0.628	0.53
CWS task errors				−.122	−1.842	0.07
Psychological health domain						
Block 1	0.294	0.079				
Physical activity				.244	3.733	< 0.0001***
Educational status				.106	1.611	0.11
Block 2	0.325	0.074	0.019			
Physical activity				.190	2.661	0.008**
Educational status				.085	1.261	0.21
MMSE score				.055	0.773	0.44
VSWM task score				.044	0.606	0.54
VWM task score				.022	0.301	0.76
CWS task errors				−.019	−.279	0.78
SS task errors				−.054	−.805	0.42
GNG task errors				−.055	−.816	0.42
Social relationships domain						
Block 1	0.135	0.014				
Educational status				.135	2.089	0.04*
Block 2	0.180	0.024	0.014			
Educational status				.107	1.611	0.11
VSWM task score				.123	1.853	0.06
Environment domain						
Block 1	0.246	0.052				
Physical activity				.214	3.214	0.001***
Marital status				−.073	−1.106	0.27
Block 2	0.372	0.112	0.078**			
Physical activity				.109	1.567	0.12
Marital status				−.038	−.569	0.58
MMSE score				.197	2.843	0.005**
VSWM task score				.004	.056	0.96
VWM task score				.103	1.478	0.14
CWS task errors				−.026	−.389	0.70
SS task errors				−.110	−1.709	0.09
Total QoL						
Block 1	0.344	0.107		.272	4.030	< 0.0001***
Physical activity				−.052	−.805	0.42
Marital status				.115	1.787	0.07
Educational status						
Block 2	0.390	0.126	0.034			
Physical activity				.202	2.854	0.005**
Marital status				−.011	−.170	0.86
Educational status				.077	1.175	0.24
MMSE score				.107	1.551	0.12
VSWM task score				.095	1.312	0.19
VWM task score				.034	.495	0.62
CWS task errors				−.067	−1.002	0.32

Significance value *p < 0.05, **p < 0.01 , ***p < 0.001

## Discussion

Our study included physically independent participants in RACFs with relatively normal cognitive function. Results revealed that participants scored highest in the domain of environment in QoL and least in the domain of social relationships in QoL. This may be due to elderly people in RACFs in Sri Lanka being mostly satisfied with the surroundings they were living in. It might have provided more opportunity to engage in spiritual and recreational activities away from family responsibilities.

Several studies have been done in different settings using various tools to assess the QoL of elderly people [33–36]. Sri Lanka has no previously published literature which assesses QoL of elderly people living in RACFs. A study conducted to assess QoL among community dwelling elderly people in Sri Lanka [35] has shown “home and neighbourhood” had the highest score which was similar to social relationships in our study that scored the least. These disparate results may be due to the difference in living arrangements, such as elderly people in care facilities being away from their usual relationships.

An Indian study found a difference in QoL domain scores between elderly people in community and institutions. Similar to our findings, they showed elderly people in institutions had scored the least in the domain of social relationships in QoL [36]. Furthermore, studies done with community elderly who had scored the highest in social relationships [37] and physical health [38], with least in psychological health [38] and physical health domains [37] in QoL, were mentioned. Although in our study, the environment domain scored the highest and social relationships domain scored the least in QoL, a study in Brazil has insisted on contradictory results [17]. Studies done with community elderly in other countries show different levels of QoL experienced by community dwelling elderly as moderate QoL [33] and good QoL [34].

A Brazilian study done with community dwelling elderly has shown a correlation between physical health domain and performance in executive function tasks and MMSE score. Their explanation was that better physical health contributes to better performance of cognitive tasks, whereas, better physical health may contribute to autonomy and independent living which may improve cognitive functioning [19]. However, Schaie and Wills argue that it may be the better cognitive health that plays a protective role against physical loss [39]. Although the Brazilian study has shown a moderate positive correlation between MMSE score and physical health, our study has demonstrated a poor correlation. In our study, each of MMSE, VSWM, VWM scores and SS, GNG and CWS task errors were correlated with psychological health. Beckert et al., [19], reflected a correlation of attention with psychological health which was not assessed in our study. This may be because people with better memory and interference control feel better psychological health.

MMSE, VSWM, VWM scores and SS, CWS task errors correlate with environment domain. This may be because better cognitive abilities perceive the living environment as an enhanced one. Other studies support this finding reflecting a correlation between environment domain and performance of executive function tasks [19] and MMSE score [17, 19], the explanation being that living in an enriched environment helps to maintain higher levels of cognitive abilities [19]. Our study showed weak correlation between MMSE score and environment domain similar to the other studies [17, 19].

Social relationship domain correlated only with VSWM score. This could be explained as those who have better memory perceive better relationships despite arguments with others. Our study did not show a significant correlation between MMSE score and social relations. Perera et al., [17] and Beckert et al., [19] also have not shown a significant correlation between MMSE score and social relations. The total QoL was correlated with MMSE, VSWM, VWM scores and CWS task errors. This finding corroborates a previous study which found correlation with MMSE score and executive functions [19]. Thus, older people with higher cognition may perceive higher life satisfaction and QoL. Moreover, those who have better memory and inhibitory control may perceive higher life satisfaction and QoL due to less interference with others.

Davis et al., [20] has shown an independent association between WM and health related QoL but not with inhibition. They have suggested further research on contribution of response inhibition to health related QoL to understand it better. In our study, interference control was associated with total QoL and three domains of QoL, except social relationships domain. The ability to inhibit ongoing responses with psychological and environment domain and ability to inhibit prepotent responses with psychological health reflect association with perceived QoL.

Although MMSE, WM scores and response inhibition task errors were correlated at statistically significant level with different domains of QoL, they were weak correlations. Hence we thought to control for covariates and to look for the existence of this relationship. For covariates, we selected educational status, marital status and physical activity as previous literature has shown a significant effect of these factors on QoL [22–24]. When controlled for the covariates, among cognitive and executive variables, only MMSE score became a predictor of environment domain of QoL. WM and IC were not predictors of QoL when controlled for covariates. The level of variance was low.

In the domain of physical health, the introduction of cognitive and executive functions only explained an additional 5.7% of variance after controlling for covariates and in environment domain of QoL, it only explained an additional 7.8% of variance after controlling for covariates. This may be because there might be other factors which affect the QoL of the elderly people dwelling in RACFs which are more important than cognitive and executive functions. Exploring these factors will assist in initiation of measures to improve QoL of elderly people in RACFs. Although physical activity was considered as a covariate, it was a predictor of total QoL and domains of physical and psychological health of QoL, which demands further explanations. Physical activity can have a positive effect on physical function and mental health in elderly people. Confidence in physical function that arises from physical activity could have contributed for this [22].

Obtaining a sample of physically independent elderly people with normal cognitive functioning might be the reason for poor correlations and for cognitive and executive functions not being the predictors of QoL. This might be different if we include elderly people with cognitive impairment in the sample. Hence, we recommend future studies with both samples using appropriate instruments. With population ageing, as there is a demand for increase in long term care of elderly in various forms [40], we suggest future studies to be further focused on the QoL of these populations.

Our study has several limitations. We could not conclude a causal relationship between level of cognition and performance of executive functions with QoL due to cross sectional design. The other limitation is the small sample size, which might affect the strength of true associations. We feel that future studies with larger sample size are needed to confirm the findings.

## Conclusion

Cognition and executive functions weakly and significantly correlated with different domains of QoL of elderly people dwelling in RACFs. Only the level of cognition measured by MMSE was a predictor of the domain of environment of QoL and executive functions were not predictors of total QoL and domains of QoL in elderly people with normal cognitive functions dwelling in RACFs.

## Abbreviations

CWS: Colour Word Stroop
EFs: Executive Functions
GNG: Go/No-Go
IC: Inhibitory control
IPAQ: International Physical Activity Questionnaire
MMSE: Mini Mental State Examination
QoL: Quality of life
RACFs: Residential aged care facilities
SD: Standard Deviation
SPSS: Statistical Package for Social Sciences
SS: Stop Signal
VSWM: Visuo-spatial Working Memory
VWM: Verbal Working Memory
WHOQoL-Bref: World Health Organization Quality of Life Bref
WM: Working memory
